# Molecular Epidemiology of Rubella Virus Strains Detected Around the Time of the 2012–2013 Epidemic in Japan

**DOI:** 10.3389/fmicb.2017.01513

**Published:** 2017-08-09

**Authors:** Yoshio Mori, Masahiro Miyoshi, Masayuki Kikuchi, Masao Sekine, Masahiro Umezawa, Miwako Saikusa, Yuki Matsushima, Masae Itamochi, Yoshihiro Yasui, Daiki Kanbayashi, Tatsuya Miyoshi, Kyoko Akiyoshi, Chika Tatsumi, Shuichi Zaitsu, Mayumi Kadoguchi, Noriyuki Otsuki, Kiyoko Okamoto, Masafumi Sakata, Katsuhiro Komase, Makoto Takeda

**Affiliations:** ^1^Department of Virology 3, National Institute of Infectious Diseases Tokyo, Japan; ^2^Hokkaido Institute of Public Health Sapporo, Japan; ^3^Sapporo City Institute of Public Health Sapporo, Japan; ^4^Sendai City Institute of Public Health Sendai, Japan; ^5^Ibaraki Prefectural Institute of Public Health Ibaraki, Japan; ^6^Yokohama City Institute of Public Health Yokohama, Japan; ^7^Kawasaki City Institute for Public Health Kawasaki, Japan; ^8^Toyama Institute of Health Toyama, Japan; ^9^Aichi Prefectural Institute of Public Health Nagoya, Japan; ^10^Osaka Institute of Public Health Osaka, Japan; ^11^Sakai City Institute of Public Health Sakai, Japan; ^12^Kobe Institute of Health Kobe, Japan; ^13^Shimane Prefectural Institute of Public Health and Environmental Science Shimane, Japan; ^14^Fukuoka City Institute of Health and Environment Fukuoka, Japan; ^15^Kumamoto City Environmental Research Center Kumamoto, Japan; ^16^Kumamoto City Hospital Kumamoto, Japan; ^17^Infectious Disease Surveillance Center, National Institute of Infectious Diseases Tokyo, Japan

**Keywords:** rubella virus, molecular epidemiology, genotype, epidemic, Japan

## Abstract

A nationwide rubella epidemic occurred from 2012 to 2013 in Japan, resulting in around 17,000 rubella cases and the birth of 45 infants with congenital rubella syndrome. The aim of this study was to genetically characterize the rubella viruses (RVs) circulating around the time of the epidemic in Japan. In total, 221 RV strains detected from 14 prefectures in Japan between 2010 and 2014 were sequenced in the 739 nucleotide-window region within the *E1* gene. The virus strains were chronologically and geographically characterized into groups based on phylogenetic analysis. Among the 221 strains analyzed, 192 (87%), 26 (12%), and 3 (1%) strains were classified into genotypes 2B, 1E, and 1J, respectively. The majority (*n* = 184) of the genotype 2B strains belonged to lineage 2B-L1 and shared nucleotide homology with the strains detected in Southeast and East Asian countries. Phylogenetic analyses demonstrated that at least six distinct clusters of RV strains (clusters 1–6) induced outbreaks in Japan between 2010 and 2014. Among them, strains from clusters 3, 4, and 6 circulated almost simultaneously during 2012–2013. The cluster 3 strains circulated locally, whereas strains from cluster 4 spread nationwide. The findings suggest that RVs were introduced into Japan many times from neighboring countries. The 2012–2013 epidemic was a complex of outbreaks induced by at least three clusters of RV strains.

## Introduction

Rubella is caused by infection with rubella virus (RV) and usually presents as a mild illness characterized by low-grade fever, a short-lived morbilliform rash, and lymphadenopathy (Reef and Plotkin, 2012). The most serious concern with this disease is that the infection to pregnant women early during their pregnancy may result in miscarriage, stillbirth, or infants born with birth defects known as congenital rubella syndrome (CRS).

The live-attenuated rubella vaccines available at present are highly effective in preventing and controlling CRS as well as rubella (Reef and Plotkin, 2012). In Japan, a single-dose rubella vaccination was introduced into the national immunization program, targeting girls in junior high schools in 1977 (Saitoh and Okabe, 2014). From 1989, children aged 12–72 months were able to receive measles vaccination using a domestic measles, mumps, and rubella combination vaccine, however, the vaccine was withdrawn in 1993 due to the relatively high incidence of meningitis caused by the mumps component (Ueda et al., 1995). In 1995, the targets of the rubella vaccination were changed to include boys and girls aged 12–90 months. Additionally, boys and girls in junior high schools were also included to the targets as a temporary measure. However, the vaccination coverage at this generation was lower than 60% (Ministry of Health, Labor, and Welfare, Japan, 2017). In 2006, a two-dose vaccination of 1- to 2- and 5- to 7-year-old children using a measles and rubella combination vaccine (MR vaccine) was introduced. Furthermore, to ensure immunization against both diseases among adolescents, a catch-up MR vaccination was implemented targeting two cohorts, those aged 12–13 and 17–18 years between 2008 and 2013. The assessment of population immunity against rubella as part of the National Epidemiological Surveillance of Vaccine-Preventable Diseases program showed that children and adolescents aged 2–24 years old and adult females had ≥90% population immunity against rubella as of 2012 (Tuberculosis and Infectious Diseases Control Division, Ministry of Health, Labor, and Welfare, Japan, and Infectious Disease Surveillance Center, National Institute of Infectious Diseases, 2015). However, up to 25% of adult males remained susceptible to rubella, in particular those in their 30s to 50s, because they had not received rubella vaccination by the routine immunization program. Since case-based surveillance of rubella started in 2008 in Japan, a low number of rubella cases were reported before 2011. However, the nationwide epidemic that occurred in 2012–2013, resulting in reports of approximately 17,000 rubella cases (Tanaka-Taya et al., 2013; Saitoh and Okabe, 2014; Ujiie et al., 2014; National Institute of Infectious Diseases, and Tuberculosis, and Infectious Diseases Control Division, Ministry of Health, Labor, and Welfare, Japan, 2015, 2016). Associated with this epidemic, a total of 45 CRS cases were reported in 2012–2014. This epidemic mainly affected males in their 30s to 50s, who had not received rubella vaccination by the routine immunization program, and males and females in their 20s, whose vaccination coverage was relatively low despite getting an opportunity to receive one or two dose rubella vaccination. The total of these demographic groups accounted for about 80% of the rubella patients in 2013.

RV belongs to the genus *Rubivirus* in the family *Togaviridae* (Hobman, 2013). The virion is enveloped by a lipid membrane and possesses a positive-sense single-stranded RNA genome of approximately 9.8-kb. The genome contains two open-reading frames that encode the non-structural proteins, p150 and p90, and the structural proteins, C, E2, and E1. The envelope glycoprotein E1 is involved in receptor binding (Cong et al., 2011) and membrane fusion (DuBois et al., 2013; Dube et al., 2014) and is the predominant antigen eliciting neutralizing or hemagglutination-inhibiting antibodies (Wolinsky et al., 1991).

The Global Vaccine Action Plan 2011–2020 endorsed by the World Health Assembly in 2012 indicated that measles and rubella were targeted for elimination in at least five World Health Organization (WHO) regions by 2020 (The Decade of Vaccine Collaboration, 2012). The surveillance of RV using molecular analysis is recognized to be important for characterizing circulating viruses in endemic countries, confirming the disappearance of endemic strains at the eliminating or eliminated stage, and tracing the transmission of newly imported strains. The Global Measles and Rubella Laboratory Network encourages genetic classification based on analysis of a 739-nucleotide window region within the *E1* gene for virological surveillance (Mulders et al., 2016). RV strains are classified into two large clades, 1 and 2, that are further divided into 10 (1a, 1B, 1C, 1D, 1E, 1F, 1G, 1H, 1I, and 1J) and three genotypes (2A, 2B, and 2C), respectively (WHO, 2013b). At present, most of the currently circulating wild-type RV strains belong to one of only four genotypes (1E, 1G, 1J, and 2B), with genotypes 1E and 2B RV strains being frequently detected worldwide (Abernathy et al., 2011; WHO, 2013b; Mulders et al., 2016; Rivailler et al., 2017). Sub-division of these genotypes has been proposed to improve the resolution of genetic classification (Rivailler et al., 2017).

In this study, we genetically characterized the 221 RV strains detected around the time of the 2012–2013 epidemic in Japan to provide insight into the epidemiology of these strains.

## Materials and Methods

### Nucleotide Sequencing of the 739-Nucleotide Window Region within the *E1* Gene

Between 2010 and 2014, clinical samples (including throat swab, blood, and urine) from suspected rubella and CRS patients were collected from patients in 14 prefectures (Hokkaido, Miyagi, Ibaraki, Tokyo, Chiba, Kanagawa, Toyama, Aichi, Osaka, Hyogo, Kagawa, Shimane, Fukuoka, and Kumamoto) and sent to local or national laboratories to detect RV as part of the national infectious agents surveillance program. Criteria for the sample collection from suspected cases with rubella or CRS varied by local government but all the samples sent to the laboratories were analyzed in principle. Samples positive for RV were subjected to nucleotide sequencing of the WHO-recommended 739-nucleotide window region within the *E1* gene (nucleotides 8731–9469) as follows. Briefly, the cDNA was synthesized using a commercial reverse transcription kit and random hexamers as primer and extracted viral RNA as template. The nucleotide region containing the 739-nucleotide window was amplified as two overlapping fragments by nested-PCR (fragment 1: nucleotides 8664–9129, fragment 2: nucleotides 9070–9492). For amplification of fragment 1, the first PCR primer set (E1-2F: 5′-AGC GAC GCG GCC TGC TGG GG-3′ and E1-2R: 5′-CCA GCG CGT ATG TGG AGT CC-3′) and the nested PCR primer set (E1-6F: 5′-ACA CCG TGA TGA GCG TGT TC-3′ and E1-10R: 5′-ATG TGG AGT CCG CAC TTG CG-3′) were used. For amplification of fragment 2, the first PCR primer set (E1-7F: 5′-AGC GAC GCG GCC TGC TGG GG-3′ and E1-12R: 5′-TGT GTG CCA TAC ACC ACG CC-3′) and the nested PCR primer set (E1-3F: 5′-CGG CGA GGT GTG GGT CAC GC-3′ and E1-3R: 5′-ACC CGC GCG CTC GCG CGA TC-3′) were used. After purification of these fragments, the nucleotide sequences were determined by a fluorescent dye-terminator cycle sequencing method using the primers E1-6F or E1-10R and E1-3F or E1-3R for fragments 1 and 2, respectively. The nucleotide sequences of the two fragments were assembled to obtain the whole sequence of the 739-nucleotide window region. The nucleotide sequences of RV strains determined in this study were submitted to the GenBank database under the accession numbers indicated in Supplemental Table 1.

### Phylogenic Analysis

Phylogenetic analysis of the sequence data obtained in this study, together with those of the genotype reference strains (WHO, 2013b), the proposed lineage reference strain candidates (Rivailler et al., 2017), and representative strains detected in other countries (Supplemental Tables 2, 3), was conducted using the MEGA program version 6.0.6. Phylogenetic trees were constructed by the maximum-likelihood method using the Tamura–Nei model (Tamura and Nei, 1993). The reliability of the tree at each branch node was assessed by the bootstrap method with 1,000 replicates. The genotype of RV strains was determined based on the phylogenetic tree topology constructed with the genotype reference strains (WHO, 2013b).

### Ethics Statement

According to the Law Concerning the Prevention of Infectious Diseases and Medical Care for Patients of Infections in Japan, rubella and CRS are defined as notifiable infectious diseases, and specimens from patients suspected of having rubella or CRS could be collected and tested for RVs without informed consent from the patients. The Ethics Committee of the National Institute of Infectious Diseases agreed to the publishing of this paper (No. 761).

## Results

### Genotyping of RV Strains Detected in Japan between 2010 and 2014

To characterize the RV strains circulating around the time of the 2012–2013 epidemic, the nucleotide sequences of the 739-nucleotide window region of 221 strains detected from rubella (*n* = 216) and CRS patients (*n* = 5) in 14 prefectures between 2010 and 2014 were determined and analyzed (**Tables 1**, **2**). The number of analyzed RV strains in each prefecture was not necessarily proportionate to the number of rubella and CRS cases. The clinical background was available for 97.7% of the rubella patients (*n* = 211). The distribution of them by gender and age are indicated in **Table 3**. The ratio of male was 75.6%, and that of adults (≥20-year-old) was 83.7%. These distributions were quite similar to those of the endemic mass population in 2013 (Tanaka-Taya et al., 2013; National Institute of Infectious Diseases, and Tuberculosis, and Infectious Diseases Control Division, Ministry of Health, Labor, and Welfare, Japan, 2015, 2016).

**Table 1:** Number of rubella viruses (RVs) analyzed in this study.

		Number of RVs analyzed in this study			
		
		Genotype			
			
Year	Number of rubella cases^1^	1E	1J	2B	Total
2010	87	1	1	3	5
2011	378	7	2	23	32
2012	2,386	11	0	63	74
2013	14,344	7	0	95	102
2014	319	0	0	8	8
Total	17,514	26	3	192	221


*^1^Reference: National Institute of Infectious Diseases, and Tuberculosis, and Infectious Diseases Control Division, Ministry of Health, Labor, and Welfare, Japan (2016).*

**Table 2:** Number of analyzed rubella viruses (RVs) by geographic area of detection and by year.

Geographic area	Number of RVs					
	
Prefecture	Year					
		
	2010	2011	2012	2013	2014	Total
Hokkaido^a^	0	2	1	9	1	13
Miyagi^b^	0	0	0	1	0	1
Ibaraki^c^	0	1	0	0	0	1
Tokyo^d^	0	1	0	0	0	1
Chiba^d^	0	0	1	0	0	1
Kanagawa^b^	4	12	29	35	5	85
Toyama^c^	1	0	0	0	0	1
Aichi^c^	0	1	12	20	2	35
Osaka^a^	0	6	4	6	0	16
Hyogo^b^	0	0	19	22	0	41
Kagawa^d^	0	0	1	0	0	1
Shimane^c^	0	0	0	7	0	7
Fukuoka^b^	0	8	7	2	0	17
Kumamoto^b^	0	1	0	0	0	1


*^a^Data from the public health laboratories in both the prefecture and the city designated by government ordinance. ^b^Data from the public health laboratory(ies) in the city(ies) designated by government ordinance. ^c^Data from the public health laboratory in the prefecture. ^d^Data from the National Institute of Infectious Diseases.*

**Table 3:** Distribution by age and gender of rubella patients who had possessed the rubella virus strains analyzed in this study.

Age group in years	Female	Male	Total (%)	
<1	0	1	1 (0.5)	
1–10	5	6	11 (5.3)	
11–19	10	12	22 (10.5)	
Subtotal (<19)	15	19	34 (16.3)
20–29	19	33	52 (24.9)	
30–39	7	64	71 (34.0)	
40–49	5	32	37 (17.7)	
≥50	5	10	15 (7.2)	
Subtotal (≥20)	36	139	175 (83.7)

Total (%)	51 (24.4)	158 (75.6)	209 (100)


*Data from the rubella patients unavailable for their clinical background (*n* = 7) and the CRS patients (*n* = 5) were excluded.*

Genotyping analysis indicated that these virus strains were classified into one of three genotypes, 1E, 1J, and 2B. The majority (*n* = 192; 87%) of these virus strains were of genotype 2B, with only 12% (*n* = 26) belonging to genotype 1E. The genotype 1J strains (*n* = 3) were only detected until 2011.

### Phylogenetic Tree Analysis of the Genotype 2B Strains

**FIGURE 1:**
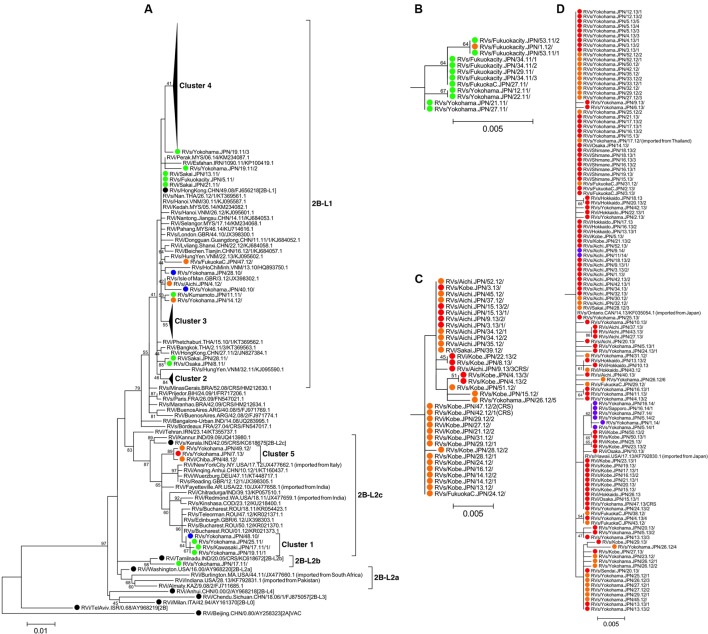
Phylogenetic tree of the genotype 2B RV strains. The phylogenetic tree was constructed using the maximum likelihood method and was based on the sequence of the 739-nucleotide-window region within the *E1* gene. Bootstrap values greater than 40% are shown adjacent to the corresponding nodes. **(A)** The entire phylogenetic tree for the genotype 2B strains. Filled triangles show clusters accumulating nodes of strains detected in Japan or exported from Japan. Panels **(B–D)** indicate the clusters 2, 3, and 4, respectively. Circles colored in blue, green, orange, red, and purple indicate strains detected in Japan in 2010, 2011, 2012, 2013, and 2014, respectively. The genotype-reference (16) and proposed lineage-reference strains (18) are indicated by black circles. The genotype 2A reference strain (RVi/Beijing.CHN/0.80/[2A]VAC) is included as an out-group.

Phylogenetic tree analysis was conducted using a dataset comprising the nucleotide sequences of the genotype 2B strains detected in Japan between 2010 and 2014 (*n* = 192) and representative 2B strains detected from 20 countries or regions (*n* = 56, Supplemental Table 2) (**Figure 1**). Almost all of the strains detected in Japan were classified into two lineages, 2B-L1 and 2B-L2c. Of the genotype 2B strains, 96% (*n* = 184) belonged to lineage 2B-L1, which was comprised strains detected in seven countries or regions (mainland China, Hong Kong, Vietnam, Malaysia, Thailand, Iran, and United Kingdom) mainly in Southeast and East Asia, suggesting that the majority of RV strains detected in Japan originated from these areas (**Figure 1A**). Lineage 2B-L2c comprised strains that originated in Europe, Africa, and Asia (**Figure 1A**).

In the phylogenetic tree, the RV strains detected in Japan predominantly formed five distinct clusters (clusters 1–5), which were supported by over 40% of bootstrap values (**Figures 1A–D**). The RV strains of clusters 1 and 2 were mainly detected in 2010 and 2011, whereas those of clusters 3, 4, and 5 were detected from 2012 to 2014. The initial strain within cluster 4 (RVs/Yokohama.JPN/17.12/) was detected from an imported case from Thailand (**Figure 1D**). Two strains detected from imported rubella cases from Japan (RVs/Ontario.CAN/14.13/ and RVs/Hawaii.USA/17.13/) belonged to cluster 4, which was consistent with the epidemiological information (**Figure 1D**).

### Phylogenetic Tree Analysis of the Genotype 1E Strains

**FIGURE 2:**
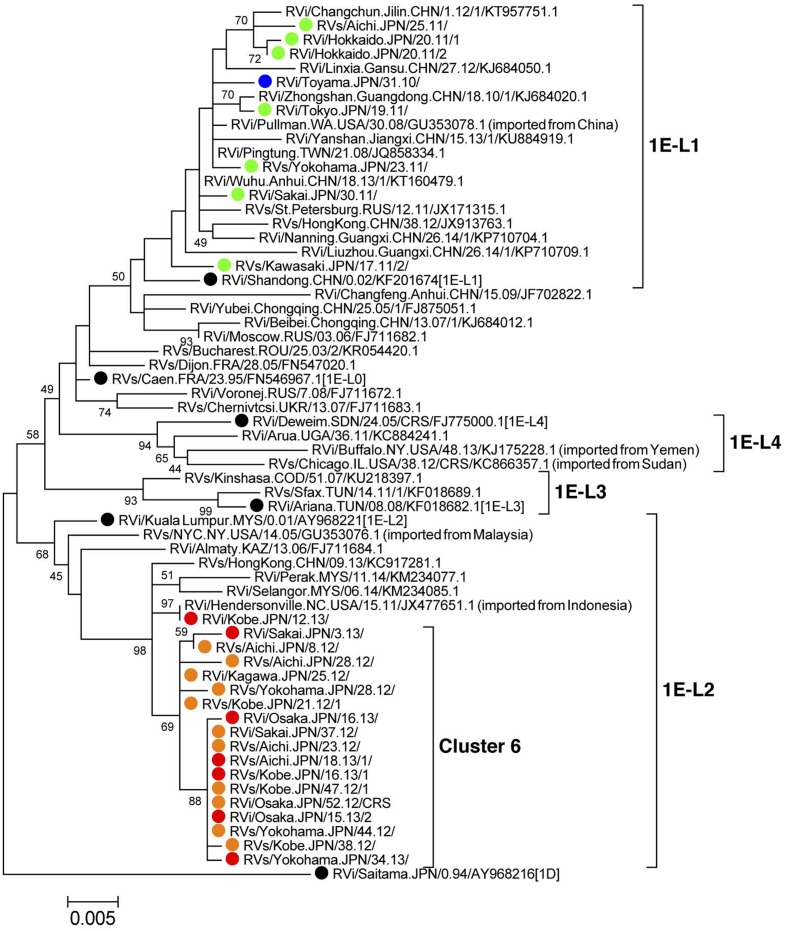
Phylogenetic tree of the genotype 1E RV strains. The phylogenetic tree was constructed using the maximum likelihood method and was based on the sequence of the 739-nucleotide-window region within the *E1* gene. Bootstrap values greater than 40% are shown adjacent to the corresponding nodes. Circles colored in blue, green, orange, and red indicate the strains detected in Japan in 2010, 2011, 2012, and 2013, respectively. The genotype-reference (16) and proposed lineage-reference strains (18) are indicated by black circles. The genotype 1D reference strain (RVi/Saitama.JPN/0.94/[1D]) is included as an out-group.

Phylogenetic analysis using a dataset consisting of nucleotide sequences from the genotype 1E strains detected in Japan between 2010 and 2013 (*n* = 26) and representative 1E strains detected in other countries or regions (*n* = 35, Supplemental Table 3) showed that the strains detected in 2010–2011 and those in 2012–2013 were classified into different lineages (**Figure 2**). The genotype 1E strains detected in Japan between 2010 and 2011 did not form distinct clusters but were included in lineage 1E-L1, which comprised strains detected in, or imported from, China, Taiwan, Hong Kong, and Russia. The genotype 1E strains detected in Japan between 2012 and 2013 belonged to lineage 1E-L2, which comprised strains detected in, or imported from, Malaysia, Hong Kong, Indonesia, and Kazakhstan, and almost all of these strains formed “cluster 6,” which was supported by 69% of the bootstrap values.

### Time Course of Detection of the Strains within Different Genetic Clusters

**FIGURE 3:**
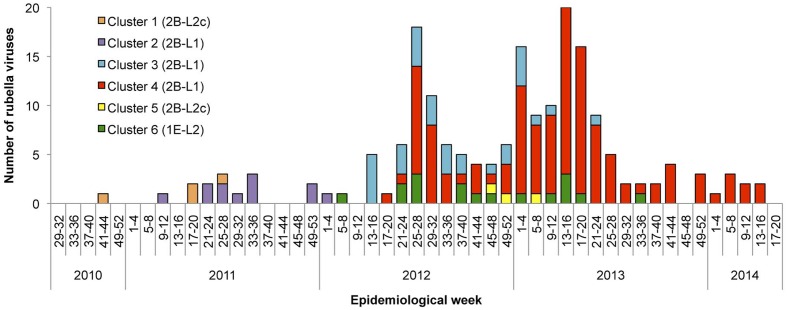
Time course of detection of RV strains by genetic group between 2010 and 2014 in Japan. The number of RV strains according to the genetic clusters defined in **Figures 1**, **2** are indicated by epidemiological weeks at disease onset or sample collection. The RV strains detected from CRS patients are not included due to a lack of information about the dates of maternal infection.

Phylogenetic analyses demonstrated that RV strains within at least six distinct genetic clusters (clusters 1–6) induced outbreaks in Japan between 2010 and 2014. **Figure 3** shows the time course of detection of these RV strains. The RV strains in clusters 1 and 2 were detected between week 48 of 2010 and week 1 of 2012. By contrast, the RV strains in the remaining four clusters were detected overlappingly between 2012 and 2013. The strains in cluster 4, the largest cluster, were continuously detected throughout a 2-year period (from week 17 of 2012 to week 16 of 2014), whereas the cluster 3 and 6 strains were detected for approximately 1 year until the middle of 2013 (from week 13 of 2012 to week 22 of 2013 and from week 8 of 2012 to week 34 of 2013, respectively). These data indicated that the 2012–2013 epidemic was a complex of outbreaks induced by several RV strains of different origins.

### Geographic Distribution of the Strains of Different Genetic Clusters

**FIGURE 4:**
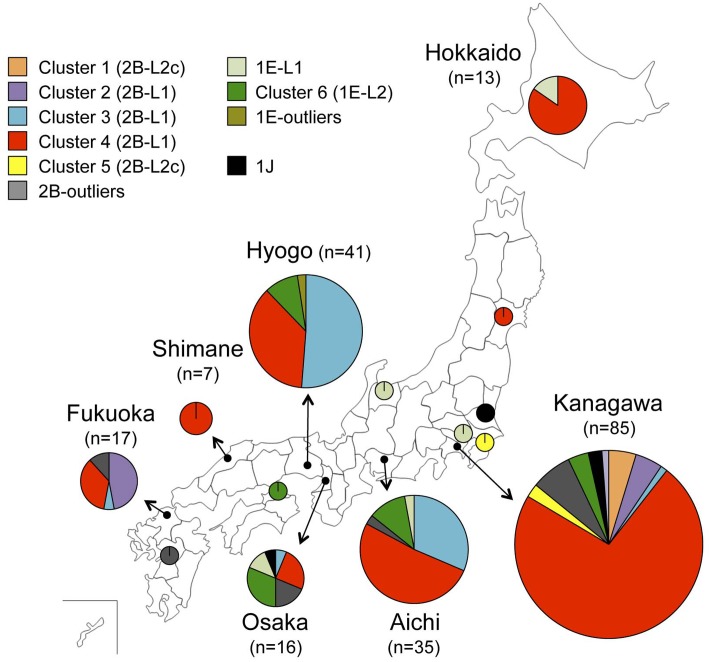
Geographic map showing the location of detection of RV strains by genetic group between 2010 and 2014 in Japan. Detection rates of the genetic clusters of RV defined in **Figures 1**, **2** by prefecture are indicated by pie charts with the sizes relating to the number of strains.

**Figure 4** shows the detection rates of the RV strains among different genetic clusters by prefecture between 2010 and 2014. The cluster 4 strains were detected in all seven prefectures where multiple RV strains were reported, suggesting that the RV strains were distributed throughout Japan. By contrast, the cluster 3 strains, the second largest group, were predominantly detected in Hyogo and Aichi prefectures and detection was limited in other prefectures, suggesting that this type of RV strain did not spread nationwide.

## Discussion

In Japan, a single dose rubella vaccination was first introduced to the routine vaccination program targeting to girls in junior high schools in 1977, however, this program could not sufficiently control endemic circulation of rubella with large epidemics occurring in cycles of about 5 years (Katow, 2004b). To overcome this situation, a single-dose rubella vaccination program and subsequently a two-dose MR vaccination program were introduced targeting both male and female children and adolescents. Following the introduction of these programs, the number of rubella and CRS cases dramatically decreased in Japan, with the lowest annual number of rubella cases (*n* = 87) being achieved in 2010. Because there has been limited genotype information on RV strains in Japan prior to 2010, it was unclear which endemic strains had previously been circulating (Katow, 2004a). The original strains used to produce the Japanese domestic vaccine, which belonged to genotype 1a, were isolated in the 1960s, and genotype 1D and 1J strains were detected in the 1990s and 2000s, respectively (Katow, 2004a; Otsuki et al., 2011; WHO, 2013b). In this study, no RV strains of genotype 1a wild-type or 1D were detected, and only three strains of genotype 1J were detected up until 2011, suggesting that transmission of these genotype strains had already been interrupted in Japan.

Most of the RV strains detected in Japan between 2010 and 2014 were classified into either genotype 2B or 1E. This is similar to the reported global situation (Abernathy et al., 2011; Tran et al., 2012; Cheng et al., 2013; WHO, 2013b; Zhu et al., 2015; Mulders et al., 2016; Rivailler et al., 2017). The global convergence of currently circulating RV genotypes makes it difficult to distinguish imported strains from endemic strains and to understand the control status of RV in each country according to genotyping data alone. To overcome this, several studies have conducted sub-division of genotypes 2B or 1E based on detailed phylogenetic analyses, although this modified classification system has not yet been standardized (Zhu et al., 2012, 2015; Cheng et al., 2013; Rivailler et al., 2017). Rivailler et al. (2017) analyzed the largest sample of worldwide RV strains investigated to date, both genetically and geographically, and proposed precise sub-grouping of genotypes 1E, 1G, and 2B, indicating reference strain candidates for each sub-group. In the present study, we analyzed and classified RV strains detected in Japan between 2010 and 2014 according to this sub-grouping. Almost all of the strains analyzed were classified into one of four different lineages, 1E-L1, 1E-L2, 2B-L1, or 2B-L2c. The majority of strains belonged to lineage 2B-L1, for which member strains were frequently reported in Southeast and East Asian countries (Tran et al., 2012; Cheng et al., 2013; Zhu et al., 2015; Rivailler et al., 2017). Before the epidemic in Japan, 2B-L1 strains were already circulating in these regions between 2006 and 2010 (Tran et al., 2012; Cheng et al., 2013) and caused a huge outbreak in 2010–2011 in Vietnam (Pham et al., 2013). It was also reported that such strains had been introduced into and spread around mainland China prior to 2011 (Zhu et al., 2015). The rapid spread of RV strains in neighboring countries preceded the introduction of this type of RV strain into Japan. In addition, there were reports that RV strains of the lineages 1E-L1 and 1E-L2 were also circulating in East and Southeast Asian countries (Cheng et al., 2013; Zhu et al., 2015; Rivailler et al., 2017). The RV strains detected in Japan between 2010 and 2014 displayed a variety of genetic backgrounds (i.e., three genotypes, four lineages, and several clusters), suggesting that these strains had been introduced from multiple sources, likely from neighboring countries. Some of countries in the WHO Western Pacific and South-East Asian regions had not introduced a rubella-containing vaccine into the national immunization program by 2014 (Grant et al., 2015).

Routine immunization coverage of the rubella-containing vaccine during the fiscal years 2011–2013 was ≥92% in two cohorts (National Institute of Infectious Diseases, and Tuberculosis, and Infectious Diseases Control Division, Ministry of Health, Labor, and Welfare, Japan, 2015) and population immunity against rubella in Japan was over 90% among children (boys and girls) and adult females in 2012 (Tuberculosis and Infectious Diseases Control Division, Ministry of Health, Labor, and Welfare, Japan, and Infectious Disease Surveillance Center, National Institute of Infectious Diseases, 2015). However, an immunization gap still remained for adult males, particularly those aged in their 30s to 50s who had not received rubella vaccination through the routine immunization program (Tanaka-Taya et al., 2013; Saitoh and Okabe, 2014; Ujiie et al., 2014). As a result of this situation, the introduction of a new RV strain might result in sporadic or small outbreaks, however, it is possible that large outbreaks, such as those seen in 2012–2013, could occur when RV spreads among adult males. This is a result of the policies of many countries including Japan to target rubella vaccination to only adolescent girls or women of childbearing age with the aim of preventing CRS in newborns (Reef and Plotkin, 2012). In these countries, it is thought that filling the immunization gap by introducing supplementary immunizations targeting susceptible populations is required for the maintenance of rubella control or elimination. Furthermore, strengthening rubella vaccination and surveillance on a global scale may be important to interrupt global circulation of these viruses.

According to the framework for verifying elimination of measles and rubella by the Strategic Advisory Group of Experts for measles and rubella (WHO, 2013a), endemic RV transmission is defined as the existence of continuous transmission of indigenous or imported RV that persists for ≥12 months in any defined geographic area. In terms of this definition, it is likely that RV strains within the clusters 3, 4, and 6 have become current endemic strains in Japan because all have been detected for ≥12 months. For verification of rubella elimination in Japan in the future, interruptions in transmission of these strains will need to be confirmed by effective surveillance systems.

The limitation of the present study is that the number of strains analyzed represented approximately 1 per 80 rubella or CRS cases and the sampling was biased to some degree by geographic area. However, our findings provide an overview of the genetic epidemiology of the 2012–2013 rubella epidemic in Japan that will be useful when devising domestic and global strategies for rubella elimination.

## Author Contributions

YoM, KK, and MT designed the study. YoM, MM, MKi, MaS, MU, MiS, YuM, MI, YY, DK, TM, KA, CT, SZ, MKa, NO, KO, and MSa determined the nucleotide sequences of the RV strains and analyzed the data. YoM and MT wrote the manuscript. All authors reviewed the manuscript.

## Conflict of Interest Statement

The authors declare that the research was conducted in the absence of any commercial or financial relationships that could be construed as a potential conflict of interest.

## Abbreviations

CRS: congenital rubella syndrome
MR vaccine: measles and rubella combination vaccine
RV: rubella virus
WHO: World Health Organization

## References

[B1] Abernathy E. S. Hubschen J. M. Muller C. P. Jin L. Brown D. Komase K. (2011). Status of global virologic surveillance for rubella viruses. *J. Infect. Dis.* 204(Suppl. 1), S524–S532. 10.1093/infdis/jir099 21666209

[B2] Cheng W. Y. Wang H. C. Liu M. T. Wu H. S. (2013). Molecular surveillance of rubella viruses in Taiwan from 2005 to 2011. *J. Med. Virol.* 85 745–753. 10.1002/jmv.23451 23417619

[B3] Cong H. Jiang Y. Tien P. (2011). Identification of the myelin oligodendrocyte glycoprotein as a cellular receptor for rubella virus. *J. Virol.* 85 11038–11047. 10.1128/JVI.05398-11 21880773 PMC3194935

[B4] Dube M. Rey F. A. Kielian M. (2014). Rubella virus: first calcium-requiring viral fusion protein. *PLoS Pathog.* 10:e1004530 10.1371/journal.ppat.1004530 PMC4256232 25474548

[B5] DuBois R. M. Vaney M. C. Tortorici M. A. Kurdi R. A. Barba-Spaeth G. Krey T. (2013). Functional and evolutionary insight from the crystal structure of rubella virus protein E1. *Nature* 493 552–556. 10.1038/nature11741 23292515

[B6] Grant G. B. Reef S. E. Dabbagh A. Gacic-Dobo M. Strebel P. M. (2015). Global progress toward rubella and congenital rubella syndrome control and elimination – 2000-2014. *MMWR Morb. Mortal. Wkly. Rep.* 64 1052–1055. 10.15585/mmwr.mm6437a5 26401958

[B7] Hobman T. (2013). “Rubella virus,” in *Fields Virology*, 6th Edn, eds Knipe D. M. Howley P. M. (Philadelphia, PA: Lippincott Williams & Wilkins), 687–711.

[B8] Katow S. (2004a). Molecular epidemiology of rubella virus in Asia: utility for reduction in the burden of diseases due to congenital rubella syndrome. *Pediatr. Int.* 46 207–213. 10.1046/j.1442-200x.2004.01866.x 15056254

[B9] Katow S. (2004b). Surveillance of congenital rubella syndrome in Japan, 1978-2002: effect of revision of the immunization law. *Vaccine* 22 4084–4091. 10.1016/j.vaccine.2004.03.055 15364460

[B10] Ministry of Health Labor Welfare Japan (2017). *Number of Vaccinees through the Routine Immunization Program (in Japanese).* Available at: http://www.mhlw.go.jp/topics/bcg/other/5.html

[B11] Mulders M. N. Rota P. A. Icenogle J. P. Brown K. E. Takeda M. Rey G. J. (2016). Global measles and rubella laboratory network support for elimination goals, 2010-2015. *Wkly. Epidemiol. Rec.* 91 240–246. 10.15585/mmwr.mm6517a3 27156255

[B12] National Institute of Infectious Diseases and Tuberculosis Infectious Diseases Control Division Ministry of Health Labor and Welfare Japan (2015). Rubella and congenital rubella syndrome in Japan, as of June 2015. *Infect. Agents Surveill. Rep.* 36 117–118. 10.7883/yoken.JJID.2014.195

[B13] National Institute of Infectious Diseases and Tuberculosis Infectious Diseases Control Division Ministry of Health Labor and Welfare Japan (2016). Measles and rubella/congenital rubella syndrome in Japan, as of March 2016. *Infect. Agents Surveill. Rep.* 37 59–60.

[B14] Otsuki N. Abo H. Kubota T. Mori Y. Umino Y. Okamoto K. (2011). Elucidation of the full genetic information of Japanese rubella vaccines and the genetic changes associated with in vitro and in vivo vaccine virus phenotypes. *Vaccine* 29 1863–1873. 10.1016/j.vaccine.2011.01.016 21251900

[B15] Pham V. H. Nguyen T. V. Nguyen T. T. Dang L. D. Hoang N. H. Nguyen T. V. (2013). Rubella epidemic in Vietnam: characteristic of rubella virus genes from pregnant women and their fetuses/newborns with congenital rubella syndrome. *J. Clin. Virol.* 57 152–156. 10.1016/j.jcv.2013.02.008 23481444

[B16] Reef S. Plotkin S. (2012). “Rubella vaccine,” in *Vaccine*, 6th Edn, eds Plotkin S. A. Orenstein W. Offit P. (London: Saunders), 688–717.

[B17] Rivailler P. Abernathy E. Icenogle J. (2017). Genetic diversity of currently circulating rubella viruses: a need to define more precise viral groups. *J. Gen. Virol.* 98 396–404. 10.1099/jgv.0.000680 27959771 PMC5797949

[B18] Saitoh A. Okabe N. (2014). Recent progress and concerns regarding the Japanese immunization program: addressing the “vaccine gap”. *Vaccine* 32 4253–4258. 10.1016/j.vaccine.2014.06.022 24951864

[B19] Tamura K. Nei M. (1993). Estimation of the number of nucleotide substitutions in the control region of mitochondrial DNA in humans and chimpanzees. *Mol. Biol. Evol.* 10 512–526. 8336541 10.1093/oxfordjournals.molbev.a040023

[B20] Tanaka-Taya K. Satoh H. Arai S. Yamagishi T. Yahata Y. Nakashima K. (2013). Nationwide rubella epidemic–Japan, 2013. *Morb. Mortal. Wkly. Rep.* 62 457–462. PMC4604843 23760185

[B21] The Decade of Vaccine Collaboration (2012). *Global Vaccine Action Plan 2011-2020. [Webpage on the Internet].* Available at: http://www.who.int/immunization/global_vaccine_action_plan/GVAP_doc_2011_2020/en/

[B22] Tran D. N. Pham N. T. Tran T. T. Khamrin P. Thongprachum A. Komase K. (2012). Phylogenetic analysis of rubella viruses in Vietnam during 2009-2010. *J. Med. Virol.* 84 705–710. 10.1002/jmv.23199 22337313

[B23] Tuberculosis and Infectious Diseases Control Division Ministry of Health Labor and Welfare Japan and Infectious Disease Surveillance Center National Institute of Infectious Diseases (2015). “Chapter 5-Rubella,” in *Proceedings of the Annual Report 2012 National Epidemiological Surveillance of Vaccine-Preventable Diseases*, Tokyo, 148–153.

[B24] Ueda K. Miyazaki C. Hidaka Y. Okada K. Kusuhara K. Kadoya R. (1995). Aseptic meningitis caused by measles-mumps-rubella vaccine in Japan. *Lancet* 346 701–702. 10.1016/S0140-6736(95)92311-X 7658837

[B25] Ujiie M. Nabae K. Shobayashi T. (2014). Rubella outbreak in Japan. *Lancet* 383 1460–1461. 10.1016/S0140-6736(14)60712-1 24766958

[B26] WHO (2013a). Framework for verifying elimination of measles and rubella. *Wkly. Epidemiol. Rec.* 88 89–99. 23540051

[B27] WHO (2013b). Rubella virus nomenclature update: 2013. *Wkly. Epidemiol. Rec.* 88 337–343. 24040673

[B28] Wolinsky J. S. McCarthy M. Allen-Cannady O. Moore W. T. Jin R. Cao S. N. (1991). Monoclonal antibody-defined epitope map of expressed rubella virus protein domains. *J. Virol.* 65 3986–3994. 1712855 10.1128/jvi.65.8.3986-3994.1991 PMC248828

[B29] Zhu Z. Cui A. Wang H. Zhang Y. Liu C. Wang C. (2012). Emergence and continuous evolution of genotype 1E rubella viruses in China. *J. Clin. Microbiol.* 50 353–363. 10.1128/JCM.01264-11 22162559 PMC3264136

[B30] Zhu Z. Rivailler P. Abernathy E. Cui A. Zhang Y. Mao N. (2015). Evolutionary analysis of rubella viruses in mainland China during 2010-2012: endemic circulation of genotype 1E and introductions of genotype 2B. *Sci. Rep.* 5:7999 10.1038/srep07999 PMC4303870 25613734

